# Determinants of university students' intention to use generative AI tools for personalized English learning: mediating effect of flow experience and moderating effect of personal innovativeness

**DOI:** 10.3389/fpsyg.2026.1728820

**Published:** 2026-05-05

**Authors:** Li Wang, Ping Deng

**Affiliations:** 1 School of Foreign Languages, Hezhou University, Hezhou, China; 2 School of Foreign Languages, Hunan International Economics University, Changsha, China

**Keywords:** flow experience, generative AI tools, personal innovativeness, personalized English learning, UTAUT2

## Abstract

**Introduction:**

Generative AI technology offers an efficient, engaging, and personalized learning experience for language learning. To encourage the utilization of generative AI tools in language study, this study has expanded the Unified Theory of Acceptance and Use of Technology 2 (UTAUT2) model to pinpoint determinants shaping university students' willingness to take these tools for personalized English learning, evaluate the mediating influence of flow experience, and analyze the moderating role of personal innovativeness.

**Methods:**

A survey was conducted among university students using a convenience sampling method, from which 386 valid questionnaires were collected for subsequent analysis. Structural equation modeling was employed to analyze the collected data with the aid of SmartPLS 4.0 software.

**Results:**

The findings indicated that performance expectancy, effort expectancy, hedonic motivation, and habit significantly affect students' behavioral intentions. Flow experience takes a partially complementary mediating role in the connections between performance expectancy, hedonic motivation, habit, and behavioral intention. Notably, the research also uncovered that personal innovativeness acts as a moderator in the link between hedonic motivation and behavioral intention. This means that for students with a higher level of personal innovativeness, the positive association between hedonic motivation and behavioral intention is stronger.

**Discussion:**

The research results provide practical implications for multiple stakeholders: for students, they can make more informed decisions to choose suitable technological tools to maximize language learning performance; for language instructors, they can better integrate new technological tools into teaching practices; for GenAI tool designers, they can develop more effective, engaging, and user-friendly products that align with university students' needs and preferences for language learning. The extended UTAUT2 model also enriches the theoretical understanding of technology acceptance in the context of personalized language learning with GenAI.

## 1 Introduction

Generative artificial intelligence (GenAI) has brought about significant changes to education, particularly in the realm of language study (Hong, 2023; Luo and Zou, 2025). By leveraging advanced algorithms and vast datasets, GenAI tools can customize learning experiences, cater to individual requirements, and boost the efficiency of educational activities (Moshayedi et al., 2022). GenAI tools are highly appropriate for personalized language learning since they can deal with individual differences by identifying the unique needs and preferences of each student, thereby optimizing the learning content, pace and activities based on individuals' language proficiency levels and preferences (Kasneci et al., 2023). Specifically, the application of GenAI tools into language study offers personalized, interactive, and adaptive learning experiences for learners (Ho, 2024). GenAI tools, including ChatGPT, Deepseek, and Doubao, have been designed to generate language similar to that of humans, create customized language learning content, provide constant feedback, and offer interactive learning experiences (Kristiawan et al., 2024). The efficiency of GenAI applications in personalized English learning (PEL) has been reflected in the acquisition of vocabulary, refinement of speaking skills, enhancement of listening abilities, improvement in reading comprehension, honing of writing techniques, and mastery of translation skills (Alshumaimeri and Alshememry, 2024). For instance, AI-driven vocabulary trainers can tailor word lists according to individual's proficiency level and areas of interest, guaranteeing that the learning process is both efficient and interesting (Ngo, 2024). Moreover, GenAI's capacity to simulate conversations and offer immediate corrections can significantly improve speaking and listening skills (Pondelíková and Luprichová, 2025). By engaging in virtual dialogues with AI-driven characters, students can practice their pronunciation, intonation, and fluency in a risk-free environment (Muniandy and Selvanathan, 2024). Similarly, AI-powered writing assistants can analyze and improve written work, offering insights into grammatical errors, stylistic choices, and coherence, thereby refining writing skills (Apriani et al., 2025; Wang, 2025). Regarding the role of GenAI tools in English reading, Shin and Lee (2023) demonstrate that they can be used to create English reading tests with fluency and naturalness of expression. Furthermore, GenAI's role in translation and interpretation is pivotal, as it provides immediate and accurate translations, allowing students to understand and compare different languages and dialects (Sa'adah et al., 2025). In the context of artificial intelligence, PEL refers to the process of using GenAI tools to dynamically generate customized content, provide real-time feedback, and continuously optimizing the learning path based on the individual needs, language proficiency, learning styles, and goals of learners (Kristiawan et al., 2024; Liu et al., 2024; Moradi, 2025). It emphasizes a learner-centered approach to create a tailor-made language learning experience by recognizing the differences in learning pace, interest preferences, and cognitive styles among individuals and adjusting learning content, methods, and progress accordingly, making learning more efficient and meaningful (Luo and Zou, 2025; Qu and Wu, 2024; Sa'adah et al., 2025). GenAI tools are of great importance and effectiveness in language learning as they can personalize learning experiences, adapt to individual needs, enhance overall educational efficacy, simulate natural language learning processes, and significantly improve various language skills like vocabulary acquisition, speaking, listening, reading, writing, and translation (Liu and Ma, 2024; Xu and Thien, 2025). Moreover, the easy accessibility and high flexibility of GenAI tools have facilitated remote and hybrid learning environments, enabling personalized language learners to study English at any time and place (Shafiee Rad, 2024). In order to enhance language learning efficiency and learning experience, it is vital to examine the determinants that would shape students' propensity to take GenAI tools for personalized language learning.

With the increasing application of GenAI into education, numerous studies have explored its potential applications, roles, benefits, opportunities, challenges, limitations, implications, and future directions (Adeshola and Adepoju, 2023; Cheng, 2023). A lot of research has taken the unified theory of acceptance and use of technology 2 (UTAUT2) to investigate how users adopt GenAI tools in education (Almousa and AbuSa'aleek, 2025; Gökçearslan et al., 2025; Polyportis and Pahos, 2024), especially in language learning (Liu and Ma, 2024; Xu and Thien, 2025). Furthermore, considering various research backgrounds, some studies have integrated variables related to demographic information, personal traits, affective, and psychological factors to extend UTAUT2 framework (Strzelecki and and ElArabawy, 2024; Wu et al., 2025; Zheng et al., 2024). University students, as digital natives, have been raised up in an era abundant with modern technologies and are perpetually encountering new innovations, their consistent exposure and acquaintance with these technologies often fosters a novel mode of thinking (Mertala et al., 2024). Their perspectives on new technologies and behaviors toward new technology adoption help understand their attitude and behavior toward language learning. Personal Innovativeness (PI) represents a distinct attribute demonstrated by individuals in their engagement with innovative technologies or concepts (Agarwal and Prasad, 1998). A number of researches have tested the role of PI on users' propensity to accept new technologies within education (Al-Adwan and Al-Debei, 2024; Gökçearslan et al., 2025). Nevertheless, few research have done to investigate the moderating function of PI on the correlation between precedents and students' willingness to take GenAI tools for PEL.

Previous research has shown that incorporating GenAI tools into language learning settings promotes a state of flow by offering immediate feedback, adjusting task difficulty to match the proficiency levels of individual learners, and creating interactive and engaging environments that keep learners focused and interested (Foroutan Far and Taghizadeh, 2024; Li et al., 2021; Xiao, 2025). Flow experience (FE) is an optimal state of experience that occurs when an individual perceives a harmonious equilibrium between their own capabilities and the challenges inherent in an activity that is intrinsically engaging (Csikszentmihalyi, 1975). Csikszentmihalyi's (1990) illustration of this concept holds significant relevance for second language acquisition, highlighting the necessity of aligning task complexity with learners' competencies to facilitate the attainment of FE. GenAI tools, when employed in personalized language study, demonstrate a great congruence with the elements of FE as delineated by Csikszentmihalyi (1975). The application of GenAI tools can empower personalized language learning by setting clear objectives and providing adaptive challenges that match learners' skills with tasks (Zhai et al., 2024). Furthermore, interactive features help foster deep engagement. When students adopt GenAI tools for personalized language learning, the GenAI tools provide engaging and immersive experiences for students, which strengthens their usage behavior. FE captures students' affective experiential mechanism, which explains the psychological process linking the antecedents to behavioral intention (BI). In this way, FE is integrated into the UTAUT2 framework to delve into its role as a mediator in the connection between precedents and students' propensity to utilize GenAI tools for PEL. Furthermore, recognizing the diversity in individuals' openness and inclination toward adopting novel ideas and technologies, this study incorporates PI into the UTAUT2 framework as a moderator to capture the individual—difference of students' willingness to take GenAI tools for PEL. The integration of FE and PI into the UTAUT2 framework provides a more comprehensive understanding of GenAI adoption for PEL by capturing two complementary perspectives: FE captures a universal psychological process that explains how students adopt GenAI tools through experiential perceptions; whereas PI acts as an individual—difference boundary condition that identifies for whom the adoption effects are stronger by considering their individual differences in embracing new technologies. By simultaneously incorporating FE and PI into the UTAUT2 framework, the model explains not only why students adopt GenAI for PEL but also for whom the effects are stronger, thereby offering a more comprehensive and nuanced account of adoption behavior.

## 2 Theoretical framework and hypotheses development

### 2.1 Theoretical underpinnings: UTAUT2

The UTAUT2 model introduces seven key determinants influencing users' BI toward technology adoption (Venkatesh et al., 2012). It explains over 70% of users' intention toward technology acceptance, making it particularly effective during initial adoption phases (Venkatesh et al., 2003). The model's capacity for explanation extends far beyond its practical applications in everyday life, a fact clearly demonstrated by its insightful predictions regarding the use of diverse new technologies in universities (Habibi et al., 2024; Hu et al., 2025; Strzelecki, 2024). The evidence from higher education stands as a robust testament to its comprehensive explanatory prowess. This capability not only demonstrates its versatility but also highlights its effectiveness in more complex and specialized scenarios. Given UTAUT2's robust predictive capabilities, this theory was utilized to explore the determinants shaping students' willingness to take GenAI tools for PEL.

In applying UTAUT2 to more accurately predict the utilization of new technologies, researchers can integrate appropriate predictors that align with specific research contexts. This involves considering diverse national backgrounds, age groups, personalities, and advancements in new technologies (Venkatesh et al., 2012). Numerous studies have expanded the UTAUT2 model through integrating additional variables, including perceived risk or threat, attention focus, attitudes toward technology, and PI, to predict students' use of modern educational technologies across various academic contexts (Haq et al., 2025; Moradi, 2025; Parveen et al., 2024; Rana and Rai, 2025; Strzelecki, 2024; Yakubu et al., 2025). Subhani et al. (2025) and Tang et al. (2025) have explored how self-efficacy, learning value, and habit act as mediators in the connection between precedents and students' willingness to take GenAI tools for educational applications. Meanwhile, a number of other scholars have investigated the moderating influences of age, gender, prior experience, academic year, motivation, information accuracy, and academic integrity on the link between the UTAUT2 components and students' adoption of modern technologies in universities (Foroughi et al., 2024; Grassini et al., 2024; Parveen et al., 2024; Subhani et al., 2025; Zheng et al., 2024). While there is an increasing trend of taking UTAUT 2 to research the application of GenAI tools within educational contexts, studies that address how personalities and affective factors affect students' utilization of these tools for PEL remain limited. Considering that university students, who are digital natives, have been raised during the age of digital products and continue to be surrounded by an environment driven by innovation. In order to capture this individual-difference boundary condition, the study tries to examine students' PI as a moderator for the connection between precedents and their willingness to take GenAI tools for PEL. Testing the moderating effect of PI will help explain for whom the model relationships are stronger within the UTAUT2 model. Additionally, the use of new technologies excites as well as challenges young people, which create FE. The perception of this experience helps explain the affective–experiential mechanism why students intend to adopt GenAI for PEL. Then, the research incorporates FE into the UTAUT2 model to test its mediating function on the link between precedents and students' willingness to take GenAI tools for PEL. Based on UTAUT2 model, understanding students' personality traits and their affective experiential perception would help them more effectively choose GenAI tools to match with their requirements and preferences for PEL.

### 2.2 Hypotheses development

#### 2.2.1 Hypotheses on the link between predictors and B1

Performance expectancy (PE) is the perceived technical usefulness, specifically the extent to which new technologies can enhance performance (Venkatesh et al., 2003). Within the UTAUT2 framework, PE acts as a direct predictor of user's decision toward technology acceptance (Venkatesh et al., 2012). Previous research manifested that PE positively impacts students' willingness to take GenAI tools in education settings (Dietrich and Grassini, 2025; Gao, 2023; Strzelecki et al., 2024). In this study, PE refers to university students' adoption of GenAI tools to enhance the efficiency of PEL and complete academic tasks or activities more effectively. For example, students can use GenAI tools to generate interesting stories to assist them in memorizing the target words. They can also use GenAI tools to help them correct grammar, polish sentences, and expand topics to improve their writing skills. BI generally assesses individual's desire to perform certain actions (Ain et al., 2015). In this context, it represents students' readiness to take GenAI tools for PEL. When students perceive these tools as efficient and useful for PEL, they are more intended to use them. Consequently, the hypothesis can be put like:

*H1: PE positively impacts students' intention to take GenAI tools for PEL*.

Effort expectancy (EE) indicates the degree to which individuals can use new technologies without difficulty or engage smoothly with the intended technologies (Venkatesh et al., 2012). Studies have demonstrated that EE serves as an important predictor for the utilization of GenAI in university education (Parveen et al., 2024; Rana and Rai, 2025; Strzelecki, 2024). In this study, students can systematically learn the functions and operation skills of GenAI tools to use them efficiently. For example, students can learn to input prompt words precisely for targeted learning content. When students want to create English conversation scenarios, they can specify parameters such as difficulty level, topic, and interaction style in the prompt to generate dialogues that match their current learning stage. By mastering such skills, students can not only reduce the time and energy but also make the learning process more efficient and tailored to their individual needs, then they are more inclined to embrace and make use of these tools for PEL. Therefore, it can be hypothesized:

*H2: EE positively impacts students' intention to take GenAI tools for PEL*.

Social influence (SI) denotes the degree to which individuals are influenced by the views of others concerning their intentions to engage in specific behaviors, particularly their decision to embrace technological advancements (Venkatesh et al., 2012). Factors rooted in social cognition, like taking into account the perspectives of significant individuals, have the capacity to forecast individuals' propensity to take new technologies (Wilson et al., 2021). Prior to making decisions to take new technologies, individuals frequently engage in interactions with one another and are susceptible to being swayed by the viewpoints of others, which subsequently prompts them to undertake particular actions (Venkatesh et al., 2003). Within this study, SI mainly originates from the opinions of students' friends, classmates, teachers, and peers regarding the adoption of GenAI tools for PEL. Studies have substantiated that SI exerts positive effect on university students' willingness to take GenAI technologies across diverse activities (Tang et al., 2025; Yakubu et al., 2025). Consequently, the hypothesis can be made:

H3: *SI positively impacts students' intention to take GenAI tools for PEL*.

Facilitating condition (FC) primarily considers whether individuals can obtain sufficient technology, infrastructure, as well as the requisite support and resources for the convenient application of new technologies (Venkatesh et al., 2012). These conditions include facilities, tools, technologies, equipment, and assistance required for using new technologies (Saxena, 2017). To ensure individuals can conveniently utilize new technologies, FC implies a seamless relationship between providers and recipients of new technologies (Dwivedi et al., 2017). Limited resources, incomplete information, delayed support, and lack of assistance may impede university students' adoption of new technologies for study. In this study, GenAI tools can be customized according to specific learning scenarios, such as integrating real-time feedback mechanisms for writing exercises or interactive language practice modules for oral practice, which help student get necessary resources and technological support. When students perceive that there is adequate support for the use of GenAI tools, they are more inclined to take them for PEL (Grassini et al., 2024). Hence, we hypothesize:

*H4: FC positively impacts students' intention to take GenAI tools for PEL*.

Price value (PV) is the balance between the perceived benefits that users derive from employing new technologies and the monetary amount they are prepared to expend (Venkatesh et al., 2003). When the perceived benefits surpass the associated costs of adopting a new technology, users consider it valuable. Several studies have revealed that PV positively impacts students' propensity to take GenAI tools into their educational endeavors (Parveen et al., 2024; Strzelecki et al., 2024). Within this research, PV can be characterized as the mental evaluation of the trade-off between the advantages acquired from utilizing GenAI tools for PEL and the corresponding costs, time, and effort expended. When students perceive that the benefits outweigh the investment required for using GenAI tools, they are more inclined to dedicate more time and effort to take these tools for PEL. Therefore, we can hypothesize:

*H5: PV positively impacts students' intention to take GenAI tools for PEL*.

Hedonic motivation (HM) is the sense of enjoyment and pleasure that individuals gain from utilizing new technologies, which takes a pivotal role in deciding to adopt technologies (Venkatesh et al., 2012). In this study, students can use GenAI tools to generate English learning games such as word puzzles, role-playing dialogues, or story creation challenges, turning dull vocabulary memorization and grammar practice into an engaging activity. The engagement and immersion brings enjoyable experience (Davis et al., 1989). These emotional responses shape users' attitudes and their readiness to engage with technological products, with those offering enjoyment generally receiving greater acceptance (Subhani et al., 2025). Foroughi et al. (2024) and Habibi et al. (2024) have confirmed that HM has a notable impact on students' willingness to take GenAI tools for academic purpose. Drawing on this evidence, it is reasonable to suggest that students who can get pleasure and happiness from the use of GenAI tools for PEL are more inclined to take these tools. Consequently, we hypothesize:

*H6: HM positively impacts students' intention to take GenAI tools for PEL*.

Habit (HB) reflects the extent of habitual or automatic behavior when using new technologies (Venkatesh et al., 2003). These unconscious and automatic behaviors emerge from prior behavioral experiences, contributing to the cultivation of a relatively consistent state (Venkatesh et al., 2003). HB is formed through continuous repetition or practice, representing a cognitive commitment to specific behaviors that develop gradually yet resist change (Murray and Häubl, 2007). As an important factor influencing the acceptance of new technologies, HB takes a vital role in using GenAI technologies in university education (Subhani et al., 2025; Tang et al., 2025). In this research, if students consistently use GenAI tools for PEL, including online vocabulary memorization, practice of speaking, listening, writing, and translating skills, they will gradually develop habitual positive behaviors for using them in PEL. Consequently, the following hypothesis can be put forward:

*H7: HB positively impacts students' intention to take GenAI tools for PEL*.

#### 2.2.2 FE mediates the link between precedents and BI

FE refers to a state of optimal experience with strong concentration and immersion in activities (Csikszentmihalyi, 1975). It is characterized by a equilibrium between task demands and individual abilities, well-defined objectives, instant feedback, task-centric attention, a high level of control, diminished self-awareness, and enjoyment (Abuhamdeh, 2020). When individuals are completely immersed in digital activities, they can get online FE (Hoffman and Novak, 2009). GenAI tools, including Doubao, Liulishuo, and Deepseek, provide students with a feeling of empowerment and mastery, ultimately enhancing language learning experience and performance (Simarmata et al., 2025). The perceived improved performance, usefulness, and efficiency of new technologies has been confirmed to significantly enhance FE (Moon and Lee, 2022). The perceived ease of utilizing GenAI tools for language study demonstrates their intuitive and user-friendly nature (Venkatesh et al., 2003). This simplicity in cognitive activities enhances the probability of attaining a flow state (deMatos et al., 2021). Additionally, both interaction with those around us and the accessibility of resources can provide timely guidance and support, thereby reducing frustration and enhancing FE (Simarmata et al., 2025). With regard to HM, the perceived enjoyment is a significant determinant of FE (Csikszentmihalyi, 1975). Furthermore, individuals generally expect the value of their usage experience to surpass the amount of money they invest (Bilgihan et al., 2014). The perception of practical value can evoke a significant emotional response from individuals (Jones et al., 2006). Thus, it is reasonable to propose that perceived PV can affect FE (Kim and Thapa, 2018). Finally, the automatic and habitual integration of new technology into daily routines can significantly help users achieve a state of optimal concentration and engagement, reaching a state of flow (Gao, 2023). This affective experiential state derived from using GenAI tools for PEL positively predicts students' attitude and behavior (Simarmata et al., 2025). Besides, previous study has confirmed that FE is a crucial predictor for users' willingness to take a new technology (Gao, 2023). Based on the preceding information, it can be hypothesized that:

*H8: FE serves as a mediator in the link between (a) PE, (b) EE, (c) SI, (d) FC, (e) HM, (f) PV, (g) HB and BI*.

#### 2.2.3 PI moderates the link between precedents and BI

Personal innovativeness (PI) refers to the disposition toward embracing new ideas and technologies (Agarwal and Prasad, 1998). It represents the open-minded attitude toward the adoption of technological advancements (Herzallah et al., 2025). Individuals with a higher PI exhibit a stronger inclination to accept technological advancements, whereas those with lower PI tend to resist such changes (Al-Adwan and Al-Debei, 2024). Previous studies have indicated that individuals with PI prefer to adopt new technologies, which stem from their curiosity about the world, adventurous spirit, enthusiasm for gathering information, and willingness to embrace new ideas with an open mindset in the face of uncertainties (Brusch and Rappel, 2020). These attributes form a powerful internal drive that motivates users with high levels of PI to try emerging technologies (Soliman et al., 2019). A plethora of investigations have stressed the vital role of PI in shaping the acceptance of ChatGPT in university education (Rana and Rai, 2025; Strzelecki et al., 2024). It is crucial to acknowledge that students are a diverse group, influenced by a multitude of factors (Foroughi et al., 2024). Being a personality trait, PI has exhibited substantial explanatory power in accounting for individual differences in technology acceptance (Foroughi et al., 2024; Senali et al., 2023). Herzallah et al. (2025) posited that PI serves as an empowering element, intensifying the link between precedents and students' propensity to take new technologies, thereby fostering stronger positive associations. With different levels of propensity to explore novel technologies, PI acts as a moderator that strengthens or lessens the interplay between precedents and BI (Alkawsi et al., 2021). Accordingly, It can be proposed that higher PI level will strengthen the link between the precedents and students' willingness to take GenAI tools for PEL. Therefore, the following hypothesis can be made:

*H9: PI moderates the impact of (a) PE, (b) EE, (c) SI, (d) FC, (e) HM, (f) PV, and (g) HB on BI*.

**Figure 1:**
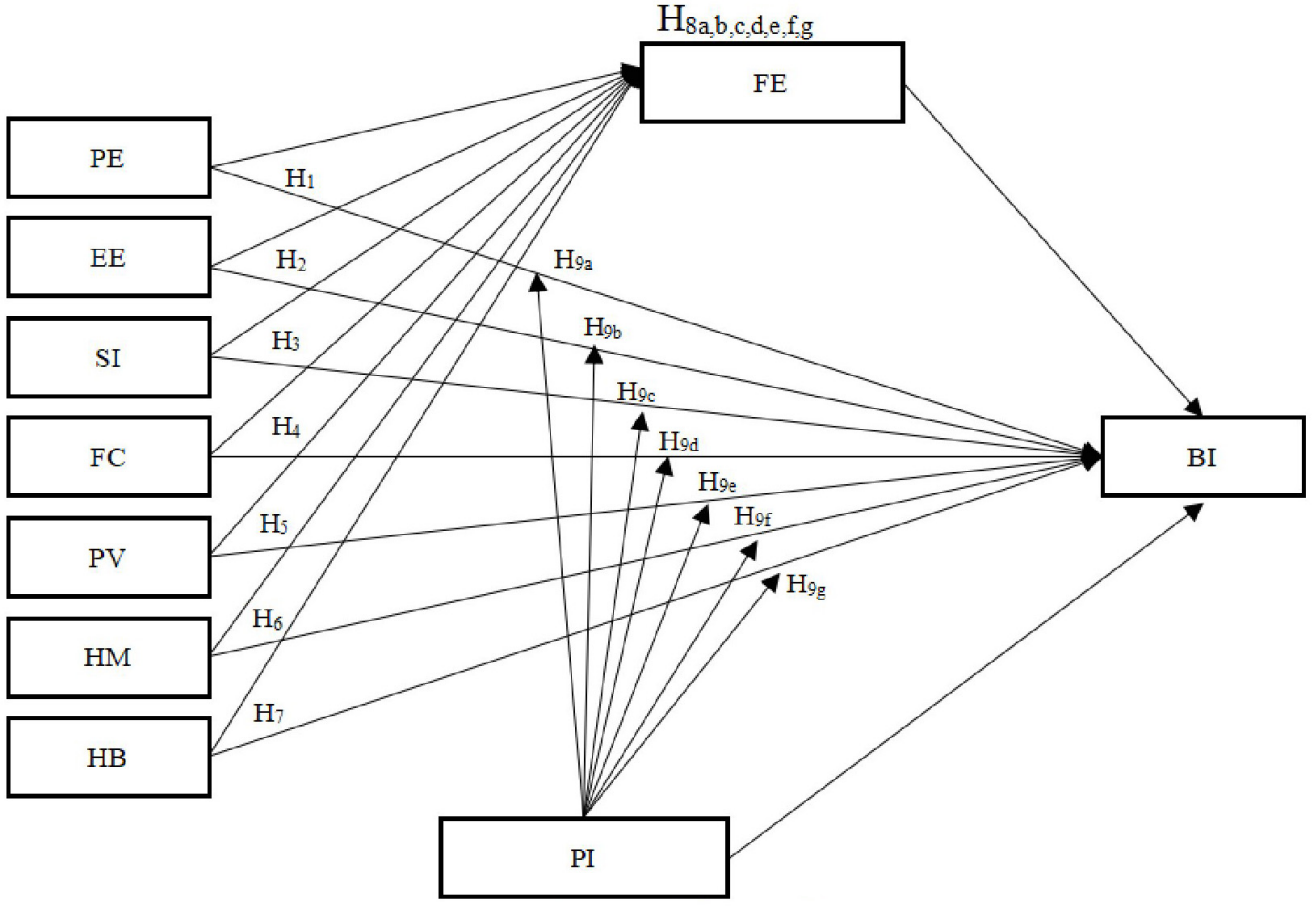
The research framework.

Building upon the aforementioned hypotheses and UTAUT2 framework, this study incorporates FE and PI into the model. The research not only investigates how precedents influence students' willingness to take GenAI tools for PEL but also examines the mediating function of FE and the moderating role of PI. The conceptual framework shows in Figure 1.

## 3 Research design

### 3.1 Measurement scales

In this study, the research model encompasses ten constructs, with a cumulative total of 30 valid measurement items (refer to Table 1). Measurement items for PI and FE derived from Farooq et al. (2017) and Kim and Jae (2019), respectively. For the remaining constructs, the measurement items were adapted from research conducted by Ain et al. (2015) and Venkatesh et al. (2012). To guarantee the items' reliability, reverse translation was employed. In order to verify that the items accurately captured their intended conceptual meanings, the questionnaire's face validity and content validity were assessed by college English teachers and experts in GenAI technology. Based on their feedback, the clarity of some statements was improved by refining the wording and sentence structure. Furthermore, 60 participants were selected through purposive sampling for the pilot study. The formal questionnaire employed the five-point Likert scale (1 = strongly disagree and 5 = strongly agree).

**Table 1:** Testing results for reliability and validity.

Construct	Measurement items	Loading	CA	CR	AVE
PE	PE1 GenAI tools are useful for PEL.	0.914	0.919	0.949	0.860
	PE2 GenAI tools help me accomplish English related activities more quickly.	0.935			
	PE3 GenAI tools increases efficiency of PEL.	0.932			
EE	EE1 GenAI tools are user-friendly for PEL.	0.890	0.872	0.921	0.795
	EE2 It is easy for me to learn how to take GenAI tools for PEL.	0.897			
	EE3 The instructions for the GenAI tools are understandable and clear.	0.888			
SI	SI1 Classmates advise me to use GenAI tools for PEL.	0.830	0.801	0.881	0.712
	SI2 Friends advise me to use GenAI tools for PEL.	0.848			
	SI3 Instructors advise me to use GenAI tools for PEL.	0.852			
FC	FC1 I can obtain sufficient resources to utilize GenAI tools for PEL.	0.858	0.808	0.886	0.722
	FC2 I possess the knowledge to utilize GenAI tools for PEL.	0.860			
	FC3 Support is available when difficulties arise with using GenAI tools for PEL.	0.831			
PV	PV1 GenAI tools are priced reasonably.	0.885	0.810	0.887	0.724
	PV2 GenAI tools offer great value for the cost.	0.835			
	PV3 At the present price, GenAI tools offer a great worth.	0.832			
HM	HM1 I feel fun using GenAI tools for PEL.	0.818	0.780	0.869	0.688
	HM2 I enjoy using GenAI tools for PEL.	0.835			
	HM3 Using GenAI tools for PEL is very entertaining.	0.835			
HB	HB1 Using GenAI tools for PEL has become a habit.	0.903	0.877	0.924	0.802
	HB2 It's habitual for me to take GenAI tools to finish PEL tasks.	0.880			
	HB3 Taking GenAI tools for PEL has become an automatic behavior.	0.903			
FE	FE1 When using GenAI tools for PEL, I feel the excitement of exploring.	0.894	0.837	0.902	0.754
	FE2 When using GenAI tools for PEL, I feel time passes quickly.	0.873			
	FE3 When using GenAI tools for PEL, I am deeply absorbed.	0.837			
PI	PI1 I like to explore new functions of digital advancements for PEL.	0.849	0.842	0.905	0.761
	PI2 I am eager to experiment new features of GenAI tools for PEL.	0.868			
	PI3 I am usually the first among my peers to take technological advances for learning.	0.898			
BI	BI1 I plan to continue taking GenAI tools for PEL in future.	0.921	0.905	0.941	0.841
	BI2 I would take GenAI tools for PEL in future.	0.900			
	BI3 I will regularly take GenAI tools for PEL in future.	0.929			

CA, Cronbach's Alpha; CR, composite reliability; AVE, average variance extracted.

### 3.2 Data collection

The research focused on university students in the Guangxi Zhuang Autonomous Region, China, as the target population. To gather data, convenience sampling was utilized via Tencent Questionnaire from April 8 to 18, 2025. Prior to administering the questionnaire, students were asked, “Are you willing to fill out this questionnaire about students' willingness to use GenAI tools for PEL?” to ensure their willingness to participate. Screening questions, such as “Have you previously used GenAI tools for PEL?” were posed to ensure that only students who had used GenAI tools for PEL were included in this study. An informed consent form was presented to students, making them aware that they possess the right and freedom to refuse the research without facing any unfavorable consequences, and assuring them that all their information is confidential. The research gathered a total of 568 questionnaires. After removing the invalid ones, there were 386 valid questionnaires left, the response rate is 68%. In Table 2, demographic statistics showed 179 males (46.4%) and 207 females (53.6%), with nearly equal gender distribution. In term of academic year, freshmen were 20.5% (79 participants), sophomores 25.4% (98 participants), juniors 29.0% (112 participants), and seniors 25.1% (97 participants). As for major, 36.5% (141 participants) were from arts and humanities, 29.3% (113 participants) were from science, and 34.2% (132 participants) were from engineering. The majority of participants reported spending more than 5 h per week using GenAI tools for PEL. Given that the sample size was tenfold greater than the number of measurement items, the 386 samples were deemed sufficient for further data analysis (Hair et al., 2017).

**Table 2:** Participants' demographic information (*N* = 386).

Measure	Category	Number	%
Gender	Male	179	46.4
	Female	207	53.6
Academic year	Freshmen	79	20.5
	Sophomores	98	25.4
	Juniors	112	29.0
	Seniors	97	25.1
Major belongs to	Arts and humanity	141	36.5
	Science	113	29.3
	Engineering	132	34.2
Frequency or weekly time spent using GenAI tools for PEL	Less than 2 h	37	9.6
	3–4 h	82	21.2
	5–6 h	136	35.2
	More than 6 h	131	33.9

## 4 Data analysis and results

Given the complexity of the research model with mediating and moderating variables, partial least squares structural equation modeling (PLS-SEM) was taken to analyze data. PLS-SEM is powerful in examining both the mediating and moderating effects within complex research frameworks (Hair et al., 2017). Data analysis was performed using IBM SPSS 22 (IBM Corporation, Armonk, NY, USA) and SmartPLS 4.0 (SmartPLS GmbH, Bonn, Germany) software packages.

### 4.1 Common method variance

Pearson correlation analysis was carried out to verify that no serious common method variance existed. Table 3 shows that the maximum correlation coefficient (0.708) is observed between FE and PI. The correlation coefficients for other construct pairs range from 0.036 to 0.687. When the Pearson correlation coefficient between construct pairs remains below 0.9, it suggests the absence of common method bias (Bagozzi et al., 1991).

**Table 3:** Pearson correlation analysis.

Construct	PE	EE	SI	FC	PV	HM	HB	PI	FE
PE	1								
EE	0.585^**^	1							
SI	0.148^**^	0.216^**^	1						
FC	0.497^**^	0.453^**^	0.172^**^	1					
PV	0.132^**^	0.171^**^	0.036	0.08	1				
HM	0.605^**^	0.470^**^	0.097	0.466^**^	0.082	1			
HB	0.692^**^	0.503^**^	0.06	0.442^**^	0.130^*^	0.538^**^	1		
PI	0.623^**^	0.564^**^	0.092	0.412^**^	0.103^*^	0.653^**^	0.613^**^	1	
FE	0.619^**^	0.464^**^	0.125^*^	0.474^**^	0.120^*^	0.636^**^	0.607^**^	0.708^**^	1
BI	0.657^**^	0.575^**^	0.146^**^	0.439^**^	0.140^**^	0.628^**^	0.631^**^	0.651^**^	0.687^**^

Correlation is significant at the 0.05 level with one asterisk(^*^) and at the 0.01 level with two asterisk(^**^).

### 4.2 Evaluation of measurement model

Considering that all constructs are reflective, the first step tests Cronbach's Alpha and composite reliability to confirm internal consistency. Table 1 displays that the Cronbach's Alpha values range between 0.780 and 0.919, while the composite reliability scores span from 0.869 to 0.949. Both metrics surpass the threshold of 0.7 yet remain below 0.95, signifying that each construct within the model possesses internal consistency reliability (Hair et al., 2017). Then, factor loadings for all items are tested to check whether the items are good measurement for each construct. Table 1 shows that factor loadings for all constructs lie between 0.818 and 0.935, exceeding the 0.7 benchmark (Hair et al., 2017). It's clear that each construct has good item reliability in this research. Next, the average variance extracted (AVE) is examined for each construct's convergent validity. Table 1 shows that AVE for the constructs ranges from 0.688 to 0.860, all surpassing the 0.5 criterion (Hair et al., 2017). More than half of the items' variance can be explained by each construct, which implies that all constructs had convergent validity based on the standards outlined by Hair et al. (2017).

After that, the Fornell-Larcker Criterion is adopted to calculate constructs' square root of AVE for assessing discriminant validity. Table 4 displays that the diagonal values of each construct significantly exceed their correlation coefficients with other constructs in any column or row, which confirms the constructs have discriminant validity. Furthermore, Heterotrait–Monotrait (HTMT) ratio is evaluated to check whether constructs are truly distinct from each other (Henseler et al., 2015). It evaluates the correlation among constructs by comparing the mean of all correlations among different constructs (Hair et al., 2017). Table 5 reveals that all correlation values of HTMT are below 0.85, and 95% confidence intervals (CI) exclude the value of 1 for all pairs of constructs (Henseler et al., 2015). Together, these results provide solid evidence that all the constructs possess strong discriminant validity.

**Table 4:** Discriminant validity with Fornell–Larcker criterion.

Construct	BI	EE	FC	FE	HB	HM	PE	PI	PV	SI
BI	**0.917**									
EE	0.576	**0.892**								
FC	0.44	0.457	**0.85**							
FE	0.688	0.468	0.473	**0.869**						
HB	0.633	0.505	0.443	0.61	**0.896**					
HM	0.657	0.493	0.47	0.654	0.556	**0.829**				
PE	0.659	0.587	0.497	0.621	0.694	0.622	**0.927**			
PI	0.652	0.564	0.41	0.707	0.613	0.686	0.623	**0.872**		
PV	0.143	0.181	0.084	0.121	0.128	0.087	0.131	0.107	**0.851**	
SI	0.149	0.222	0.173	0.127	0.061	0.111	0.15	0.094	0.042	**0.844**

Bold-faced diagonal values are square roots of AVE.

**Table 5:** Discriminant validity with Heterotrait–Monotrait ratio (at the 95% CI).

Construct	BI	EE	FC	FE	HB	HM	PE	PI	PV
EE	0.647 [0.556, 0.728]								
FC	0.513 [0.391, 0.617]	0.539 [0.423, 0.638]							
FE	0.788 [0.709, 0.852]	0.543 [0.439, 0.637]	0.577 [0.449, 0.618]						
HB	0.708 [0.624, 0.778]	0.575 [0.487, 0.652]	0.523 [0.411, 0.62]	0.707 [0.605, 0.789]					
HM	0.746 [0.67, 0.814]	0.57 [0.463, 0.656]	0.588 [0.48, 0.683]	0.785 [0.693, 0.864]	0.65 [0.559, 0.731]				
PE	0.72 [0.65, 0.781]	0.653 [0.574, 0.719]	0.575 [0.47, 0.669]	0.704 [0.596, 0.79]	0.772 [0.707, 0.827]	0.714 [0.631, 0.787]			
PI	0.745 [0.664, 0.811]	0.658 [0.552, 0.746]	0.497 [0.364, 0.608]	0.841 [0.771, 0.899]	0.714 [0.616, 0.794]	0.803 [0.734, 0.861]	0.708 [0.618, 0.784]		
PV	0.163 [0.071, 0.26]	0.205 [0.12, 0.305]	0.1 [0.039, 0.161]	0.146 [0.06, 0.245]	0.154 [0.061, 0.255]	0.103 [0.04, 0.198]	0.152 [0.062, 0.248]	0.126 [0.052, 0.215]	
SI	0.17 [0.069, 0.284]	0.258 [0.151, 0.369]	0.216 [0.112, 0.325]	0.154 [0.061, 0.265]	0.075 [0.029, 0.138]	0.122 [0.5, 0.208]	0.172 [0.076, 0.268]	0.111 [0.039, 0.222]	0.065 [0.024, 0.097]

### 4.3 Evaluation of structural model

Before the structural model evaluation, the variance inflation factor (VIF) values of all independent variables in the structure model were examined to check collinearity issue. In Table 6, all VIF values were under the threshold value of 3.3 (Diamantopoulos and Siguaw, 2006). Therefore, there is no critical issues of collinearity in this structural model.

**Table 6:** Testing results of R^2^, Q^2^, and VIF.

Construct	*R* ^2^	*Q* ^2^	VIF								
			PE	EE	SI	FC	PV	HM	HB	FE	PI
BI	0.650	0.574	2.738	1.901	1.163	1.679	1.094	2.380	2.322	2.668	2.896
FE	0.544	0.518	2.568	1.743	1.070	1.488	1.037	1.828	2.100		

To effectively evaluate the model, bootstrapping was conducted 5,000 times with a two-tailed test. The predictive power of the structural model comes first. Table 6 illustrates that the coefficient of determination (*R*^2^) for forecasting students' willingness to take GenAI tools for PEL through independent variables stands at 0.650, indicating that endogenous latent variables provide substantial explanatory power for the research model (Hair et al., 2017). The explanatory capacity of these variables for the dependent variable exceeds 60%. Then follows the model's predictive relevance. In Table 6, the *Q*^2^ value for predictive model correlation is positive (0.574), demonstrating the path model has substantial predictive relevance for students' willingness to take GenAI tools for PEL (Hair et al., 2017). As for the effect size, HM exerts the strongest influence on students' propensity to take GenAI tools for PEL among the independent variables (*f*^2^= 0.053).

After testing the hypothesized relationship, the path coefficients in Table 7 illustrate that PE, EE, HM, and HB all show positive associations with students' willingness to take GenAI tools for PEL. Specifically, HM displays the largest correlation, next comes with HB, EE, and PE. The *t*-values for all these variables exceeded 1.96, *p*-values were below 0.05, and 95% CI did not encompass zero. These findings highlight substantial positive relationships between these variables and students' willingness to take GenAI tools across all these path coefficients, thus supporting hypotheses H1, H2, H6, and H7. Conversely, SI, FC, and PV show no significant correlations. All *t*-values fall below 1.96, *p*-values surpass 0.05, and 95% CI includes zero. This suggests that SI, FC, and PV have no effect on students' inclination to take GenAI tools for PEL, consequently disproving hypothesis H3, H4, and H5.

**Table 7:** Testing results for path coefficients and hypotheses.

H	Relationships direct effect	β	*T*-value	*P*-value	95% IC	*f* ^2^	Supported or not
H1	PE → BI	0.141	2.552	0.011^*^	[0.028, 0.246]	0.021	Yes
H2	EE → BI	0.161	3.651	0.000^***^	[0.075, 0.250]	0.039	Yes
H3	SI → BI	0.015	0.429	0.668	[−0.051, 0.089]	0.001	No
H4	FC → BI	0.018	0.406	0.685	[−0.097, 0.072]	0.001	No
H5	PV → BI	0.023	0.711	0.477	[−0.040, 0.086]	0.001	No
H6	HM → BI	0.210	4.136	0.000^***^	[0.110, 0.307]	0.053	Yes
H7	HB → BI	0.157	3.152	0.002^**^	[0.062, 0.257]	0.030	Yes

^*^p < 0.05; ^**^p < 0.01; ^***^p < 0.001 (two-tails).

### 4.4 Analysis of mediating effects

Table 8 presents the outcomes of the mediating effect analysis of FE. Regarding H8a, FE demonstrates a complementary partial mediating effect in the link between PE, HM, HB, and students' willingness to take GenAI tools for PEL. Specifically, PE (β = 0.043, *T* value = 2.143, *P* value = 0.032, 95% CI = 0.006 to 0.084), HM (β = 0.092, *T* value = 3.848, *P* value = 0.000, 95% CI = 0.047 to 0.141), and HB (β = 0.061, *T* value = 2.878, *P* value = 0.004, 95% CI = 0.024 to 0.107) all affect BI through the mediating pathway of FE. All *t*-values surpass 1.96, the *p*-values fall below 0.05, and the 95% CI does not include 0, all of which signify that the mediating role is significant. These results support hypotheses H8a, H8f, and H8g. Conversely, for the mediating paths of EE → FE → BI, SI → FE → BI, FC → FE → BI, and PV → FE → BI, the results indicate that all *t*-values are less than 1.96, *P*-values above 0.05, and the 95% CI encompasses zero. This indicates that FE does not mediate these relationships, which fails to support hypotheses H8b, H8c, H8d, and H8e.

**Table 8:** Testing results of mediating effect of FE.

H	Indirect effect of FE on BI	β	*T* value	*P* value	95% CI		Mediation type	Results
H8a	PE → FE → BI	0.043	2.143	0.032	0.006	0.084	Complementary partial mediation	Yes
H8b	EE → FE → BI	0.004	0.34	0.734	−0.018	0.033		No
H8c	SI → FE → BI	0.007	0.721	0.471	−0.010	0.026		No
H8d	FC → FE → BI	0.027	1.815	0.070	−0.000	0.057		No
H8e	PV → FE → BI	0.006	0.779	0.436	−0.009	0.024		No
H8f	HM → FE → BI	0.092	3.848	0.000	0.047	0.141	Complementary partial mediation	Yes
H8g	HB → FE → BI	0.061	2.878	0.004	0.024	0.107	Complementary partial mediation	Yes

### 4.5 Analysis of moderating effects

**Figure 2:**
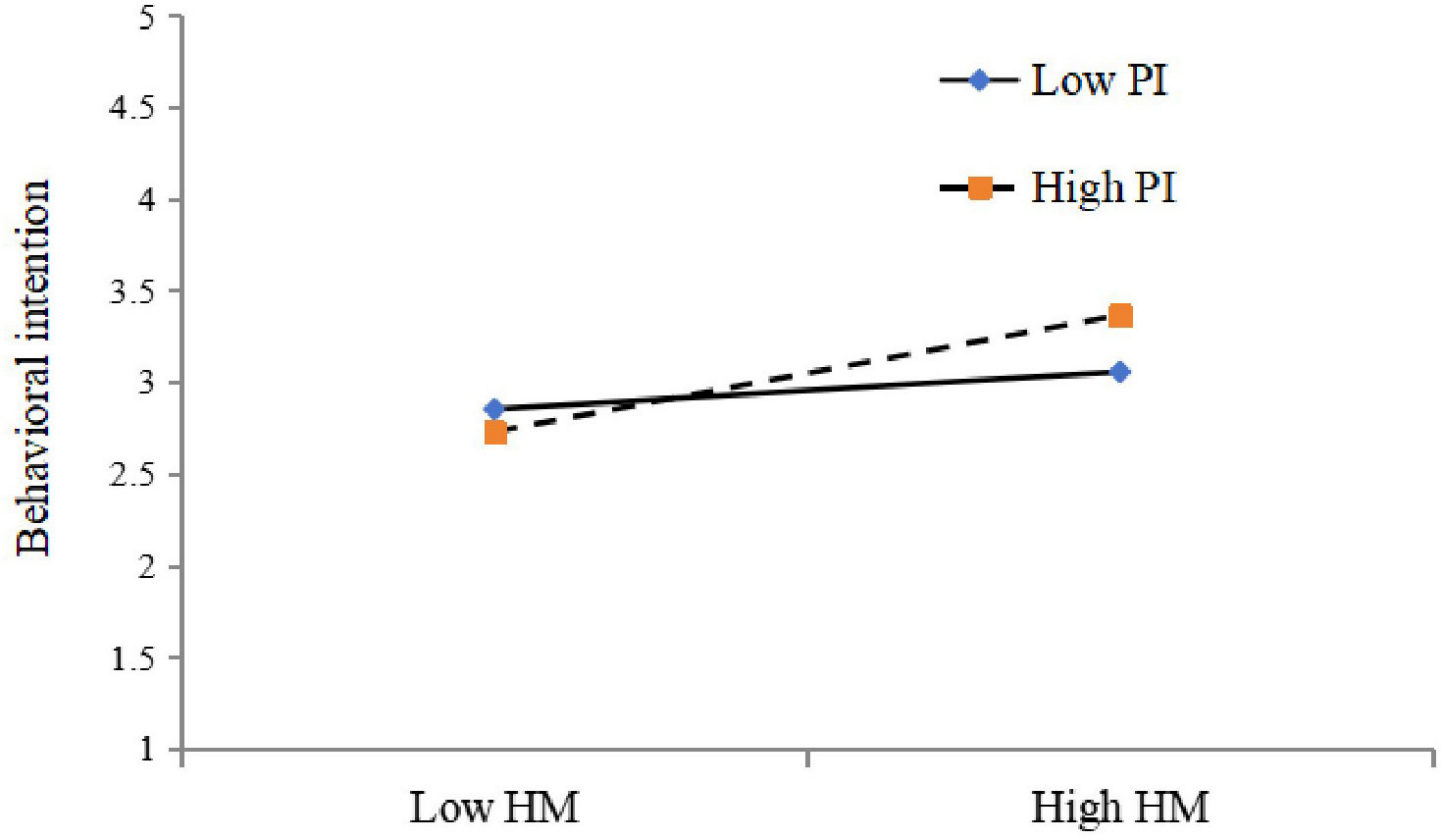
Moderating effect of PI on the HM*BI relationship.

As displayed in Table 9, among all the proposed moderating relationships in the main model path with PI, it only moderates the link between HM and students' willingness to take GenAI tools for PEL(β =0.108). The *t*-value (2.301) exceeded 1.96, with a *p*-value (0.021) under 0.05. The 95% CI excluded the zero value, indicating that PI positively moderates the link between HM and BI, thus supporting hypothesis H8f. Figure 2 visually demonstrates this moderating effect more vividly. For every additional standard deviation in PI, the slope of HM on BI increases by 0.108 standard deviations. There are substantial differences in the extent to which HM positively affects BI across various levels of PI. In particular, individuals with a higher degree of PI display a steeper slope compared to those with a lower level, demonstrating that HM has a prominent effect on BI for those with superior PI. Nevertheless, there was no moderating effect of PI on the links between other independent variables and BI. Notably, regarding the moderating effect of PI on the relationship between EE and BI, even though the *t*-value (2.046) is above 1.96 and the *p*-value (0.04) is less than 0.05, since the 95% CI includes 0, this relationship is not significant. In bootstrapping, the *p*-value and CI are calculated from empirical distribution rather than parametric distribution, which can occasionally lead to non-aligned results. For other relationships, all *t*-values are below 1.96, the *p*-values surpass 0.05, and the 95% CI includes zero. All these imply that there was no moderating effect of PI on the links between PE, EE, SI, FC, HB, and BI. Consequently, hypotheses H8a, H8b, H8c, H8d, H8e, and H8g are all rejected.

**Table 9:** Testing results of moderating effect of PI.

H	Relationships moderating effect of PI	β	*T*-value	*P*-value	95% CI	Supported or not
H9a	PI^*^PE → BI	−0.103	1.697	0.090	[−0.225, 0.012]	No
H9b	PI^*^EE → BI	−0.101	2.046	0.041	[−0.184, 0.012]	No
H9c	PI^*^SI → BI	0.068	1.659	0.097	[0.010, 0.149]	No
H9d	PI^*^FC → BI	−0.008	0.18	0.857	[−0.107, 0.071]	No
H9e	PI^*^PV → BI	0.054	1.312	0.190	[−0.027, 0.137]	No
H9f	PI^*^HM → BI	0.108	2.301	0.021	[0.001, 0.187]	Yes
H9g	PI^*^HB → BI	0.094	1.643	0.100	[−0.015, 0.210]	No

## 5 Discussion

### 5.1 Discussion on the link between precedents and BI

Drawing upon the extended UTAUT2 framework, this study has explored the determinants shaping students' propensity to utilize GenAI tools in PEL. Beyond pinpointing the determinants, the research has also evaluated the mediating role of FE and the moderating function of PI. The framework demonstrates substantial explanatory capacity concerning the variance in FE and BI. The findings reveal that PE, EE, HM, and HB exert a notable effect on students' inclination to take GenAI tools for PEL, whereas SI, FC, and PV do not significantly affect BI. FE acts as a mediator in the connections between PE, HM, HB, and BI, whereas PI only moderates the association between HM and BI.

More specifically, the present study provides evidence that PE positively affects students' willingness to take GenAI tools for PEL. This outcome supports the results of prior studies done by Liu et al. (2024) as well as Qu and Wu (2024). These studies indicated that when students perceive greater efficiency and utility in using GenAI tools for language-related tasks and assignments, their inclination to use these tools for language learning increases. A possible reason for this positive relationship, as proposed by Foroughi et al. (2024), is that GenAI tools, which are equipped with advanced natural language processing algorithms, are capable of offering students customized learning experiences. They can adjust to students' learning preferences and provide customized feedback. The positive impact of PE on BI underscores the significance of developing GenAI tools that can enhance students' perception of efficiency and utility in PEL. To achieve this, designers of GenAI tools ought to give priority to improve these tools' ability to quickly understand students' individual learning needs and preferences, for example, generating context-specific grammar exercises or tailoring vocabulary lists according to students' current proficiency level. In this way, GenAI tools can better align with students' requirements, which will increase their language learning efficiency.

The research results also demonstrate that EE significantly affects students' inclination to take GenAI tools for PEL. This result aligns with the investigations conducted by Tang et al. (2025) and Dietrich and Grassini (2025), which validates the importance of EE in forecasting students' inclination to take new technological tools in university education. The little effort needed to master GenAI tools makes them more alluring as an educational aid, enhancing students' possibility of utilizing them (Foroughi et al., 2024). Furthermore, the user-friendly nature of modern educational technology can alleviate students' cognitive burden and help them concentrate on studying rather than struggling with the technology (Chen et al., 2021). When students can easily use GenAI tools' functions to precisely match with their language learning needs, such as enlarging vocabulary and improving listening skills, they are more intended to take these tools for PEL. This suggests that designers should prioritize user experience and ensure that GenAI tools are designed with clear instructions and simple operation procedures.

Moreover, HM stands out as the most important factor influencing students' willingness to employ GenAI tools for PEL. This observation supports the findings presented by Xu and Thien (2025) and Zheng et al. (2024), who also highlighted that enjoyment is significant in students' decisions to utilize GenAI tools for language study. Such enjoyment stems from multiple sources, such as the innovative aspect of the technology and the engaging quality of the dialogue-driven interface (Strzelecki, 2024). This insight reveals that students are intended to interact with GenAI tools if they perceive the experience pleasurable, entertaining, or emotionally fulfilling. This result stresses the importance for tool designers and educators to prioritize the application of interactive, game-like, or visually engaging components to elevate the hedonic elements for these tools. For example, incorporating elements like personalized feedback, progress tracking with rewards, or interactive materials could enrich the language learning journey, thus boosting students' propensity to consistently use GenAI tools.

In the present research, HB emerged as the second most significant determinant influencing students' inclination to utilize GenAI tools for PEL. This finding reinforces prior studies by Caner-Yildirim (2025) and Strzelecki (2024), which demonstrated that frequent and sustained engagement with emerging technologies can facilitate their easy integration into academic practices. Similarly, Surya Bahadur et al. (2024) highlighted HB as a critical driver influencing students' inclination to take GenAI tools in higher education contexts. The plausible explanation could be that HB cultivates technological proficiency, thereby increasing students' propensity to repeatedly use specific tools. In order to help students form a good habit of using GenAI tools for PEL, some structured training programs, such as daily vocabulary exercises or weekly writing feedback sessions, can be designed to guide learners through regular interactions with GenAI tools. Over time, students become more adept at using GenAI tools for PEL.

In contrast, the research uncovered that SI does not serve as a predictor for students' propensity to utilize GenAI tools for PEL. The result upholds the conclusions drawn by Grassini et al. (2024), which suggest that users with a higher level of education, like university students, exhibit a lower susceptibility to external societal pressures. Nevertheless, the outcome diverges from the findings of prior studies examining learning technologies in university education (Strzelecki et al., 2024; Yakubu et al., 2025). The plausible explanation might be that the GenAI-supported PEL is highly private, individualized, and self-directed rather than relying on group behavior (Sa'adah et al., 2025). Students' decision to use GenAI tools as personalized learning tools is more likely to stem from individual or personal factors, such as their subjective assessment of its utility and effectiveness, rather than social pressure or advice from peers and teachers (Caner-Yildirim, 2025). These discrepant conclusions drawn from existing research highlight the complexity and context-dependency of factors influencing students' intention to adopt technology in educational settings. This underscores the need for future research to carefully consider the specific nature and implementation context of technology-integrated learning environments when exploring the predictive factors of students' BI.

The current study found no evidence of a positive link between FC and students' inclination to take GenAI tools for PEL, which is in consonance with the findings of Surya Bahadur et al. (2024) and Haq et al. (2025). Nevertheless, this outcome diverges from the conclusions of recent studies by Caner-Yildirim (2025) and Rana and Rai (2025), which highlighted FC as a key determinant for students' decision to take educational technologies. Such inconsistencies may stem from differences in participant demographics, the specific GenAI tools examined, or the context in which these tools were deployed (Grassini et al., 2024). In the present study, the insignificant effect of FC on BI might be attributed to the fact that the university students, being digital natives, possess a high level of proficiency with contemporary digital technologies and search engines (Mertala et al., 2024; Parveen et al., 2024). Their familiarity with new digital technologies may reduce the need for additional knowledge and support, thus having a relatively small influence on students' intention to take GenAI tools for learning (Foroughi et al., 2024; Hu et al., 2025). With sufficient access to the internet and GenAI tools, students can easily use them for study without help or support. Thus, they may not consider FC as a major factor influencing their adoption decisions in this study.

Opposite to our original hypothesis, the impact of PV on shaping students' willingness to employ GenAI tools for PEL is not significant. This finding aligns with the research from Moradi (2025) and Zheng et al. (2024), which found that PV has no effect on BI. Their plausible explanation for the lack of significance is the perceived free availability of GenAI tools. If students perceive GenAI tools as readily accessible without additional costs, their perceived impact of PV on BI may be weakened. However, this result contradicts existing literature, which posits that PV positively impacts students' inclination to take new technologies for educational applications (Strzelecki, 2024). The contradictory research results highlight the complexity of identifying the UTAUT2 factors in predicting students' BI to adopt GenAI tools. This disparity indicates the necessity of conducting further research to explore the contextual factors that would influence the relationship between PV and BI in the specific domain of technology adoption for language learning. Future studies could investigate the interplay between PV and BI in different technological and educational contexts.

### 5.2 Discussion on the mediating effect of FE

The complementary partial mediating effect of FE on the link between predictors and students' inclination to take GenAI tools for PEL reveals a nuanced dynamic. Specifically, PE, HM, and HB do not directly exert an influence on BI. Rather, their impacts are partially channeled through FE. This implies that when students view GenAI tools as capable of improving their language learning performance, obtain pleasure from using them, or develop a habitual usage tendency, these elements contribute to an enhanced state of flow during the learning process. The heightened FE, subsequently, reinforces their inclination to continue using GenAI tools for PEL. However, EE, SI, FC, and PV fail to result in a state of FE that affects BI. For educators and institutions, this suggests that designing GenAI tools with interactive features that induce flow states, such as gamified elements, instant feedback, and customized learning paths, could enhance the positive effects of PE, HM, and HB on BI. For designers, integrating FE-enhancing mechanisms (e.g., personalized challenges, progress tracking) may bridge the gap between functional attributes and user adoption.

### 5.3 Discussion on the moderating effect of PI

Concerning the moderating influence of PI, the results indicate that PI positively strengthens only the association between HM and BI, mirroring the research findings of Herzallah et al. (2025). This positive moderating effect suggests that as PI increases, the impact of HM on students' willingness to take GenAI tools for PEL becomes stronger. It may be that students with a higher level of PI are more intended to welcome new technological advancements, especially when such technologies offer hedonic benefits, such as entertainment or enjoyment. Nevertheless, PI did not exhibit moderating impacts on the links between other predictors, including PE, EE, SI, FC, PV, and HB, and users' intention to take GenAI tools for PEL. It could be that these variables are less sensitive to individual variances in PI, or other elements might assume a more prominent role in moderating these associations. For educators, this outcome highlights the significance of recognizing and nurturing students' individual innovative traits. AI tool designers should take note of the role that PI plays in facilitating technology adoption. They can design GenAI tools with attributes that appeal to innovative users, such as customized interfaces, interactive learning modules, and gamified features.

## 6 Research implication

### 6.1 Theoretical implication

This research carries substantial theoretical significance. Initially, the findings indicate that PE, EE, HM, and HB positively impact students' inclination to take GenAI tools for PEL, which offers empirical support for the effectiveness of the UTAUT2 model within the realm of university educational context. This further confirms this model's suitability in elucidating students' behavior in using GenAI learning tools, demonstrating its applicability in tertiary education, particularly in language study. Furthermore, by integrating FE as a mediator and PI as a moderator into the expanded UTAUT2 framework, this study deepens the theoretical framework for comprehending students' adoption of GenAI tools for PEL. This expansion of the UTAUT2 model enriches the theoretical framework, enabling researchers to better account for the intricate interaction of diverse variables in students' application of technological advancements for language study. It underscores the necessity of considering not only the direct determinants but also the psychological and individual differences that may influence the adoption process.

### 6.2 Practical implication

This results offer practical implications for students, designers of GenAI tool, language instructors, and educational institutions. From the perspective of students, understanding the determinants influencing their propensity to take GenAI tools for PEL enables them to make wiser choices and more informed decisions to choose technologies to align with their language learning needs. The research reveals that PE, EE, HM, and HB are key influencing elements. In order to strengthen PE, students should select GenAI tools that meet their needs based on their weak points and goals in PEL, such as improving listening skills, expanding vocabulary, enhancing oral fluency, and optimizing writing logic. For instance, if the goal is to improve listening, one can use GenAI's audio generation function to obtain audio materials of different scenarios, speeds, and accents for repeated intensive listening. During the usage process, the functions of GenAI tools should be precisely matched with students' language learning needs. To reduce the difficulty of using GenAI tools for PEL, students should systematically learn the functions and operation skills of GenAI tools. For instance, learning how to precisely input prompt words can lead to more targeted learning content. When practicing oral English, inputting *Simulate a College English Test Band 4 speaking topic “Describe a memorable experience” and provide real-time scoring and feedback on my response* is more effective than simply inputting *Practice oral English*. Additionally, students can utilize GenAI tools' customization settings to adjust the learning interface, content presentation methods, etc., to better suit their learning habits and improve usage efficiency. Considering the importance of HM in shaping students' BI, students can try using AI to generate creative English content like rap and poetry, integrating dull knowledge points into interesting forms to make learning more enjoyable. In order to develop a good habit of using GenAI tools for PEL, students should make reasonable plans for using these tools and adhere to them, gradually developing the habit of using these tools for PEL. In the plan, the content and goals for each learning session should be clearly defined, such as practicing listening on Mondays, memorizing vocabulary on Tuesdays, and doing writing exercises on Wednesdays. They can set a fixed daily learning time, such as from 7 to 8 p.m. every day, to use GenAI for PEL. Over time, using generative AI tools for English learning will become a natural behavior. Moreover, the result shows that FE mediates the association between PE, HM, HB, and BI. The mediating function of FE highlights the importance of complete immersion and engagement. To enhance FE, students can strive to create an immersive learning environment by choosing a quiet and comfortable learning space and turning off the notification alerts of electronic devices like mobile phones. During the learning process, students focus entirely on the learning content provided by the GenAI and follow its guidance to gradually delve deeper into the learning. For instance, during reading practice, students should follow the GenAI's pace, analyze the main idea, details, and logical relationships of each paragraph, and promptly ask the GenAI for explanations when encountering difficulties.

For designers of GenAI tools, it is crucial to grasp the influencing factors (PE, EE, HM, and HB) that shape university students' willingness to take GenAI tools for PEL. By doing so, they can design tools that significantly enhance English learning results (PE), are intuitive and easy to operate (EE), deliver pleasurable and captivating learning experiences (HM), and foster consistent usage through habitual patterns (HB). Moreover, the mediating influence of FE highlights the necessity of establishing an immersive and engaging learning atmosphere. GenAI tool designers ought to devise tools capable of triggering a flow state, in which students are completely engrossed in the learning journey and experiencing enjoyment and accomplishment. This objective can be realized by integrating interactive features, tailored learning routines, and instant feedback systems that keep student motivated and engaged. By considering these elements, GenAI tool designers have the potential to produce more efficient, captivating, and user-centric products that align with students' distinct requirements and preferences for PEL.

From the viewpoint of language instructors, the findings offer insights for boosting the incorporation and efficiency of GenAI tools in foreign language instruction. Initially, grasping the factors impacting students' willingness to take GenAI tools enables instructors to more efficiently integrate these technologies into language teaching process. They can leverage these factors to design more engaging and user-friendly GenAI-based language learning experiences. Furthermore, the mediating function of FE underscores the necessity of fostering an immersive and engaging educational setting. Language instructors should design GenAI-based tasks that elicit a flow state, in which learners are completely engrossed and concentrated on their current assignment. Achieving this involves setting clear goals, delivering instant feedback, and maintaining an equilibrium between task difficulty and learner proficiency. Language instructors can draw on the research findings to devise a more compelling, efficient, and tailored educational atmosphere that encourages students' inclination to take GenAI tools throughout their language learning process.

For educational institutions, the research results can inform policy-making and resource allocation. The institutions should allocate sufficient resources to support the use of GenAI tools into language study. This may include providing access to reliable AI-based language learning platforms, training on how to seamlessly take these tools into teaching process, and guaranteeing that students have the necessary devices and internet access. The mediating influence of FE underscores the significance of establishing an immersive environment for students. This goal can be attained by devising personalized learning paths that match with the specific requirements and preferences of each learner. Challenges should be appropriately balanced, neither overly simple nor excessively difficult. In this way, students can completely absorb and actively engage in the learning journey. Furthermore, the moderating impact of PI on the bond between HM and BI indicates that educational institutions should actively encourage and support innovative thinking among students.

### 6.3 Limitations and future study

This study can be further advanced from the following aspects. To begin with, the research was carried out in Guangxi Zhuang Autonomous Region in China, which limited the wide applicability of the results to other settings. Subsequent research could be undertaken in diverse cultural and educational environments to determine if the relationships identified in this study remain valid across varying educational and cultural contexts. Secondly, as this was a cross-sectional research, it could not fully reflect the changing nature of the elements under investigation. Future research could try longitudinal research to gain a thorough understanding of the relationships among the variables. For instance, future study could test the long-term impacts of using GenAI tools on students' English proficiency and evaluate whether the adoption of GenAI tools improves language skills over an extended period. Thirdly, another intriguing area for future investigation is the influence of different kinds of GenAI tools on students' learning experiences. There are numerous GenAI-based applications available, each with its own features and functions. Moreover, future study could delve into the role of other potential moderators or mediators in the technology adoption process. For instance, factors such as students' prior technology experience, individual personalities, learning styles, or language proficiency could also affect their propensity to take GenAI tools for PEL. Lastly, the UTAUT2 model used in this research doesn't include students' actual behavior, which would limit its ability to explain how intentions change into actual behaviors. Although BI is a strong predictor in UTAUT2, intentions don't always change into actual usage, especially in educational contexts. In technology based language learning contexts, students may abandon using GenAI tools because of internal and external constraints. Future research can incorporate actual behavior into this model for language learning contexts. Longitudinal data can be collected to test whether students' intentions translate into actual behaviors.

## 7 Conclusion

Grounded in the extended UTAUT2 model, this research has explored the determinants shaping students' willingness to utilize GenAI tools for PEL. Besides identifying the influential factors, the research also evaluated the mediating function of FE and the moderating role of PI. The findings indicate that PE, EE, HM, and HB affect students' willingness to take GenAI tools for PEL, whereas SI, FC, and PV have no effect on BI. FE acts as a mediator on the connections between PE, HM, HB, and BI, whereas PI only moderates the bond between HM and BI. Understanding the determinants that affect students' intention to take GenAI tools for PEL can empower students to make more informed decisions to choose suitable technological tools to match with their language learning needs and maximize language learning performance. This research results can also help GenAI tool designers to create more effective, engaging, and user-friendly products to meet university students' needs and preferences for PEL. The mediating function of FE and moderating effect of PI highlight the importance to foster an immersive and stimulating learning atmosphere and encourage innovative thinking among students. These insights can navigate the design and application of future educational technologies, guaranteeing that they are practical, engaging, and aligned with students' preferences and needs.

## Data Availability

The original contributions presented in the study are included in the article/Supplementary material, further inquiries can be directed to the corresponding author.
