# Supplementary material for: Determinants of university students' intention to use generative AI tools for personalized English learning: mediating effect of flow experience and moderating effect of personal innovativeness
Source: Front Psychol. 2026 May 5;17:1728820. doi: 10.3389/fpsyg.2026.1728820 (PMC13218346; doi:10.3389/fpsyg.2026.1728820)
Supplement: Supplementary file 1 [file Table_1.DOCX]

Chinese version of the finalized questionnaire

**调查问卷：大学生使用生成式人工智能工具进行个性化英语学习的意向影响因素：心流体验的中介效应与个人创新性的调节作用**

亲爱的同学们： 大家好！

这是一份旨在了解大学生使用生成式人工智能工具进行个性化英语学习意向的问卷。问卷采取不记名方式，全部资料仅供统计分析之用，绝不对外公开。恳请您帮忙填答，谢谢您的热心参与。

祝你 生活愉快，学习进步！

第一部分：问卷主要内容，请根据你的实际感受和情况回答下列问题，请于□中打「🗸」，谢谢！

| 构念 | 测量指标项 | 非常不同意1 | 不同意  2 | 一般  3 | 同意  4 | 非常同意 5 |
| --- | --- | --- | --- | --- | --- | --- |
| 绩效期望 | PE1 生成式人工智能工具对个性化英语学习非常有用。 | □ | □ | □ | □ | □ |
|  | PE2 生成式人工智能工具能帮助更快地完成英语任务。 | □ | □ | □ | □ | □ |
|  | PE3 生成式人工智能工具能提升个性化英语学习效率。 | □ | □ | □ | □ | □ |
| 努力绩效 | EE1 使用生成式人工智能工具进行个性化英语学习极为便捷友好。 | □ | □ | □ | □ | □ |
|  | EE2 我能够轻松学会如何使用生成式人工智能工具进行个性化英语学习。 | □ | □ | □ | □ | □ |
|  | EE3 生成式人工智能工具的操作指南简洁明了，易于理解。 | □ | □ | □ | □ | □ |
| 社会影响 | SI1 同学建议我使用生成式人工智能工具进行个性化英语学习。 | □ | □ | □ | □ | □ |
|  | SI2 朋友建议我使用生成式人工智能工具进行个性化英语学习。 | □ | □ | □ | □ | □ |
|  | SI3 老师建议我使用生成式人工智能工具进行个性化英语学习。 | □ | □ | □ | □ | □ |
| 便利条件 | FC1 使用生成式人工智能工具进行个性化英语学习时，我能够获取足够的资源来。 | □ | □ | □ | □ | □ |
|  | FC2 我具备丰富的知识储备，能够高效运用生成式人工智能工具，开展个性化的英语学习。 | □ | □ | □ | □ | □ |
|  | FC3 使用生成式人工智能工具进行个性化英语学习遇到困难时，我能够获得充分的支持。 | □ | □ | □ | □ | □ |
| 价格价值 | PV1 生成式人工智能工具的价格合理。 | □ | □ | □ | □ | □ |
|  | PV2 生成式人工智能工具的性价比很高。 | □ | □ | □ | □ | □ |
|  | PV3 以目前的价格，生成式人工智能工具提供了显著的价值。 | □ | □ | □ | □ | □ |
| 享乐动机 | HM1我认为使用生成式人工智能工具进行个性化英语学习颇具趣味性。 | □ | □ | □ | □ | □ |
|  | HM2我非常享受使用生成式人工智能工具进行个性化英语学习。 | □ | □ | □ | □ | □ |
|  | HM3使用生成式人工智能工具进行个性化英语学习非常有趣。 | □ | □ | □ | □ | □ |
| 习惯 | HB1我使用生成式人工智能工具进行个性化英语学习已成为一种习惯。 | □ | □ | □ | □ | □ |
|  | HB2我已习惯使用生成式人工智能工具来完成个性化英语学习任务。 | □ | □ | □ | □ | □ |
|  | HB3使用生成式人工智能工具进行个性化英语学习已成为一种自觉选择。 | □ | □ | □ | □ | □ |
| 心流体验 | FE1 使用生成式人工智能工具进行个性化英语学习时，我深切体会到探索的乐趣与激动。 | □ | □ | □ | □ | □ |
|  | FE2 使用生成式人工智能工具进行个性化英语学习时，时间仿佛转瞬即逝。 | □ | □ | □ | □ | □ |
|  | FE3 使用生成式人工智能工具进行个性化英语学习时，我全身心投入其中。 | □ | □ | □ | □ | □ |
| 个人创新性 | PI1 我乐于于探索数字技术进步为个性化英语学习带来的新功能。 | □ | □ | □ | □ | □ |
|  | PI2 我迫不及待想在个性化英语学习中尝试生成式人工智能工具的新功能。 | □ | □ | □ | □ | □ |
|  | PI3 我通常是同龄人中第一个采用创新技术进行个性化英语学习的人。 | □ | □ | □ | □ | □ |
| 行为意向 | BI1 我计划在将来继续使用生成式人工智能工具进行个性化英语学习。 | □ | □ | □ | □ | □ |
|  | BI2 将来我会使用生成式人工智能工具进行个性化英语学习。 | □ | □ | □ | □ | □ |
|  | BI3 今后我会定期使用生成式人工智能工具进行个性化英语学习。 | □ | □ | □ | □ | □ |

第二部分：基本信息，请于□中打「🗸」，谢谢！

性别: □男 □女

学年: □大一 □大二 □大三 □大四

专业: □艺术与人文 □理科 □工科

每周使用频率: □2小时及以下 □3-4小时 □5-6小时 □6小时以上

本问卷到此结束。

非常感谢你的耐心填答以及为将生成式人工智能工具应用于个性化英语学习的研究所做的贡献！
