# Supplementary material for: Determinants of university students' intention to use generative AI tools for personalized English learning: mediating effect of flow experience and moderating effect of personal innovativeness
Source: Front Psychol. 2026 May 5;17:1728820. doi: 10.3389/fpsyg.2026.1728820 (PMC13218346; doi:10.3389/fpsyg.2026.1728820)
Supplement: Supplementary file 2 [file Table_2.DOCX]

English version of the finalized questionnaire

### Questionnaire about [the Determinants for University Students' Intention to Take Generative AI Tools for Personalized English Learning: Mediating effect of Flow Experience and Moderating](http://en.cnki.com.cn/Article_en/CJFDTOTAL-CYYK201406017.htm) Effect of Personal Innovativeness

Dear students:

Thank you very much for taking time to fill out this questionnaire. The data you provide will be for academic use only and personal information will be kept strictly confidential. Thank you for your support and participation.

One: Main part of the questionnaire. Please tick 「🗸」 in the corresponding box □ based on your actual experience and perceptions.

| Please tick (√) in the corresponding box to express your attitude towards your intention to take generative artificial intelligence (GenAI) tools for personalized Egnlish learning (PEL). | | | | | | |
| --- | --- | --- | --- | --- | --- | --- |
| No. of items | Measurement items for performance expectancy(PE) | Strongly disagree | Disagree | Neutral | Agree | Strongly agree |
| PE1 | GenAI tools are useful for PEL. |  |  |  |  |  |
| PE2 | GenAI tools help me accomplish English related activities more quickly. |  |  |  |  |  |
| PE3 | GenAI tools increases efficiency of PEL. |  |  |  |  |  |
| No. of items | Measurement items for effort expectancy(EE) | strongly disagree | disagree | neutral | agree | strongly agree |
| EE1 | GenAI tools are user-friendly for PEL. |  |  |  |  |  |
| EE2 | It is easy for me to learn how to take GenAI tools for PEL. |  |  |  |  |  |
| EE3 | The instructions for the GenAI tools are understandable and clear. |  |  |  |  |  |
| No. of items | Measurement items for social influence (SI) | strongly disagree | disagree | neutral | agree | strongly agree |
| SI1 | Classmates advise me to use GenAI tools for PEL. |  |  |  |  |  |
| SI2 | Friends advise me to use GenAI tools for PEL. |  |  |  |  |  |
| SI3 | Instructors advise me to use GenAI tools for PEL. |  |  |  |  |  |
| No. of items | Measurement items for facilitating conditions(FC) | strongly disagree | disagree | neutral | agree | strongly agree |
| FC1 | I can obtain sufficient resources to utilize GenAI tools for PEL. |  |  |  |  |  |
| FC2 | I possess the knowledge to utilize GenAI tools for PEL. |  |  |  |  |  |
| FC3 | Support is available when difficulties arise with using GenAI tools for PEL. |  |  |  |  |  |
| No. of items | Measurement items for price value(PV) | strongly disagree | disagree | neutral | agree | strongly agree |
| PV1 | GenAI tools are priced reasonably. |  |  |  |  |  |
| PV2 | GenAI tools offer great value for the cost. |  |  |  |  |  |
| PV3 | At the present price, GenAI tools offer a great worth. |  |  |  |  |  |
| No. of items | Measurement items for hedonic motivation(HM) | strongly disagree | disagree | neutral | agree | strongly agree |
| HM1 | I feel fun using GenAI tools for PEL. |  |  |  |  |  |
| HM2 | I enjoy using GenAI tools for PEL. |  |  |  |  |  |
| HM3 | Using GenAI tools for PEL is very entertaining. |  |  |  |  |  |
| No. of items | Measurement items for habit(HB) | strongly disagree | disagree | neutral | agree | strongly agree |
| HB1 | Using GenAI tools for PEL has become a habit. |  |  |  |  |  |
| HB2 | It’s habitual for me to take GenAI tools to finish PEL tasks. |  |  |  |  |  |
| HB3 | Taking GenAI tools for PEL has become an automatic behavior. |  |  |  |  |  |
| No. of items | Measurement items for flow experience(FE) | strongly disagree | disagree | neutral | agree | strongly agree |
| FE1 | When using GenAI tools for PEL, I feel the excitement of exploring. |  |  |  |  |  |
| FE2 | When using GenAI tools for PEL, I feel time passes quickly. |  |  |  |  |  |
| FE3 | When using GenAI tools for PEL, I am deeply absorbed. |  |  |  |  |  |
| No. of items | Measurement items for personal innovativeness(PI) | strongly disagree | disagree | neutral | agree | strongly agree |
| PI1 | I like to explore new functions of digital advancements for PEL. |  |  |  |  |  |
| PI2 | I am eager to experiment new features of GenAI tools for PEL. |  |  |  |  |  |
| PI3 | I am usually the first among my peers to take innovative technologies for learning. |  |  |  |  |  |
| No. of items | Measurement items for behavioral intentions(BI) | strongly disagree | disagree | neutral | agree | strongly agree |
| BI1 | I plan to continue taking GenAI tools for PEL in future. |  |  |  |  |  |
| BI2 | I would take GenAI tools for PEL in future. |  |  |  |  |  |
| BI3 | I will regularly take GenAI tools for PEL in future. |  |  |  |  |  |

Two: Basic information. Please tick 「**🗸**」 in the corresponding box □.

Gender: □male □female

Academic year: □ freshman □sophomore □junior □senior

Major: □arts and humanity □science □engineering

Usage frequency: □less than 2 hours □3-4 hours □ 5-6 hours □more than 6 hours

That’s the end of this questionnaire.

Thank you very much for your contribution to this questionnaire or even to the integration of GenAI tools for English study. Wish you have a good day.
