# Supplementary material for: Determinants of university students' intention to use generative AI tools for personalized English learning: mediating effect of flow experience and moderating effect of personal innovativeness
Source: Front Psychol. 2026 May 5;17:1728820. doi: 10.3389/fpsyg.2026.1728820 (PMC13218346; doi:10.3389/fpsyg.2026.1728820)
Supplement: Supplementary file 3 [file Table_3.DOCX]

**Participant Consent Form参与者知情同意书**

**Name of Project:** [**The Determinants for University Students' Intention to Take Generative AI Tools for Personalized English Learning: Mediating Effect of Flow Experience and Moderating**](http://en.cnki.com.cn/Article_en/CJFDTOTAL-CYYK201406017.htm) **Effect of Personal Innovativeness**

项目名称: 大学生使用生成式人工智能工具进行个性化英语学习的意向影响因素：心流体验的中介效应与个人创新性的调节作用

Name of Researcher: Ping Deng, Wang Li

研究者: 邓萍，王丽

Dear students:

亲爱的同学：

**Please read the statements below. If after reading these statements you no longer wish to participate, please feel free to withdraw, there will be no consequences.**

**敬请阅读以下陈述。如果您在阅读之后不希望后续参与，请直接放弃，这不会有任何后果。**

1. I am over 18 years old.

我已年满18岁。

1. I confirm that I have read and I understand the participant information sheet for this study. I further confirmed that I have had the opportunity to consider the information so provided, ask questions and received appropriate answers which I deemed satisfactory.

我已阅读参与研究知情书。本人有足够机会了解这项研究的相关信息，所提问题均能得到适当且满意的解答。

1. By handing this questionnaire back to you, completed, I am giving my consent for you to use my questionnaire answers in this research study.

通过提交填妥的问卷，我同意使用我的作答来参与这项研究。

1. I understand that my participation is totally voluntary. I am free to withdraw at any time without giving any reason if I wish.

我可自由决定是否参加这项研究，如果愿意，我有权在任何时候无需任何理由选择退出此次问卷调查。

1. I agree to participate in this research.

我同意参与此项研究。

Signature签名:

Date 日期
