# Supplementary material for: Determinants of university students' intention to use generative AI tools for personalized English learning: mediating effect of flow experience and moderating effect of personal innovativeness
Source: Front Psychol. 2026 May 5;17:1728820. doi: 10.3389/fpsyg.2026.1728820 (PMC13218346; doi:10.3389/fpsyg.2026.1728820)
Supplement: Supplementary file 4 [file Table_4.DOCX]

**Participant Information Sheet 研究参与知情书**

**Name of Project:** [**The Determinants for University Students' Intention to Take Generative AI Tools for Personalized English Learning: Mediating Effect of Flow Experience and Moderating**](http://en.cnki.com.cn/Article_en/CJFDTOTAL-CYYK201406017.htm) **Effect of Personal Innovativeness**

项目名称: 大学生使用生成式人工智能工具进行个性化英语学习的意向影响因素：心流体验的中介效应与个人创新性的调节作用

Name of Researcher: Ping Deng, Wang Li

研究者: 邓萍，王丽

Dear students:

亲爱的同学：

You are invited to participate in a research to explore the determinants for university students’ intention to take generative AI tools for personalized English learning.

诚邀您参与一项研究，该研究旨在探索大学生使用生成式人工智能工具进行个性化英语学习的意向影响因素。

Before you decide on your undertaking, you need to understand the nature of this research, why it is done and what is your involvement. Please ensure that you read the following the information carefully. Consult others about this study if you wish. Please do not hesitate to ask me anything that is not clear or if you would like more information.

在您决定参与此次前，您有权知悉该研究的性质，目的以及您的参与角色。请确保仔细阅读以下信息。 如果您愿意或需要的话，也可咨询其他相关人员。如有任何疑虑或想了解更多信息，我都会尽力解答。

1. **What is the purpose of the study?** **此项研究的目的？**

Through identifying the determinants for university students’ intention to take generative AI tools for personalized English learning, the study aims to provide valuable insights for English instructors, educational institutions, and developers of educational technology on how to effectively improve the functions of such tools and implement them for language learning.

本研究通过探究大学生使用生成式人工智能工具进行个性化英语学习的意向影响因素，旨在为英语教师、教育机构以及教育技术开发者提供实用建议，帮助他们优化这些工具的功能并有效应用于语言教学。

1. **Why have I been invited? 为什么我会被邀请？**

Because you, as digital native, who have used generative AI tools for personalized English learning, have the final word in providing information about university students’ intention to take generative AI tools for personalized English learning.

作为数字原住民，您在使用生成式人工智能工具进行个性化英语学习方面经验丰富，因此关于大学生对这类工具的使用意向方面，您最有话语权。

1. **Do I have to take part? 我一定要参与吗？**

No, you are under no obligation to take part in this study. If you decide to take part and then change your mind for whatever reason, you are free to withdraw at any time without any consequences. If you can spare some time to help finish the questionnaire, I really appreciate your help.

不是的，您并没有参与这项研究的义务。如果您决心参与，但随后无论任何原因想要退出，您是有权力随时放弃的。如果您能抽出宝贵的时间帮忙填写此份研究问卷，我将非常感激您的帮助！

1. **What will taking part involve me?** **如果我愿意参与，我需要做些什么？**

If you would like to take part in this research, you will be invited to fill in a questionnaire which will ask your view about the factors that would shape your intention to take generative AI tools for personalized English learning. It will take you about three to five minutes to finish the questionnaire.

如果您愿意参与这项研究，我们将邀请您填写一份问卷，以了解您对影响使用生成式人工智能工具进行个性化英语学习意向的各类因素的看法。完成问卷预计将占用您大约三到五分钟的时间。

1. **Expenses and payment 费用与报酬**

This study is completely voluntary, and you will not receive any payment from it. Thank you for giving your time to participate in the study.

此项研究完全是自愿的，您不会从中取得任何报酬。感谢您在百忙之中参与。

1. **What are the possible disadvantages and risks of taking part? 参与此研究有任何弊端和风险吗？**

There are no potential risks associated with the study.

这项研究没有潜在风险。

1. **Will my taking part in this study be kept confidential? 我在这项研究中的作答将是保密的吗？**

Yes. All information regarding your identity and data gathered will remain confidential at all times. The data will be used only for this research to explore the determinants for university students’ intention to take generative AI tools for personalized English learning. If you are interested in this study and would like to know more about the research progress and results, findings will be shared in the aggregate only. No respondent-specific data will be shared. Please be ensured that your privacy and anonymity will be protected.

是的。所有关于您的信息在任何时候都是保密的。本次研究的数据将仅用于本研究，探析大学生使用生成式人工智能工具进行个性化英语学习的意向影响因素。如果您对本项研究感兴趣并想进一步了解研究进展和成果的话，可分享整体的研究成果，有关参与者的个人具体信息不可分享。您的隐私和匿名性将得到保护。

1. **What are the possible benefits of taking part? 参与这项研究有什么好处吗？**

There are no direct benefits from taking part in the study.  However, you will contribute to the research that tries to explore the determinants for university students’ intention to take generative AI tools for personalized English learning.

参与这项研究并没有直接的好处。但您将为探索大学生使用生成式人工智能工具进行个性化英语学习意向影响因素的研究提供支持。

1. **What will I have to do? 接下来我该做什么？**

After you have a full understanding about the research, you are invited to fill in a questionnaire to help and promote this research, please sign this consent form before go forward to the questionnaire.

当您了解该研究的相关内容后，您将被邀请填写一份问卷，在填写问卷之前，请先在参与者知情同意书上签名。

Contact for Further Information 联系信息

Researcher研究者：Deng Ping 邓萍

Cellphone number移动电话：13557842649

Email Address 电子邮箱：dengping_123@163.com

Thank you for reading this information sheet.
